# An Unmerited Slur

**Published:** 1896-09

**Authors:** 


AN UNMERITED SLUR is cast upon the Texas medical profession by the *Texas Medical News* [windmill], which says, in effect, that the Texas State Medical Association and local societies, in affiliation, embrace all that is decent or respectable in the profession of the State; or, to be exact (having said that in Polk's Directory a very large number of names are followed by an *, which means, "no report received in answer to inquiry regarding graduation"), the *News* proceeds: "This class, as a rule, represents the great unwashed medicine men who practice without medical diplomas. When, now, the members of the State Association and of the county and district associations, that are in affiliation, are placed on one side, there will be left on the other side a host of the 'unwashed' who are not eligible to membership in the State Medical Association. It is this class the editor of the TEXAS MEDICAL JOURNAL refers to when he says, 'on behalf of the great body of the profession and of himself,' the writer 'repudiates the action of that body.'"

Not so; that there are a large number of physicians put down in the Directory with an * opposite their names, no one will deny; but it does not follow, by any means, that all whose names are so entered are ineligible to membership, or that they are practicing without a diploma. And it was not to those so practicing we referred. The profession of the State is estimated at 5000; the membership in the State Association is about 400. It was the remaining 4600, who are outside of the State organization, to whom we referred as being not bound by the action of that body; and this constitutes over nine-tenths of the profession. The *News* thus casts a slur upon the "great body of the profession"—those whom it is sought to enroll in the State Association. If this isn't a case of the tail wagging the dog, we do not know where a better illustration could be found.

